# Development and validation of non‐guided bladder‐neck and neurovascular‐bundle dissection modules of the RobotiX‐Mentor® full‐procedure robotic‐assisted radical prostatectomy virtual reality simulation

**DOI:** 10.1002/rcs.2195

**Published:** 2020-11-13

**Authors:** Jan Ebbing, Peter N. Wiklund, Olof Akre, Stefan Carlsson, Mats J. Olsson, Jonas Höijer, Maurice Heimer, Justin W. Collins

**Affiliations:** ^1^ University Hospital Basel Department of Urology Basel Switzerland; ^2^ Karolinska University Hospital Department of Urology Stockholm Sweden; ^3^ Karolinska Institutet Department of Molecular Medicine and Surgery (MMK) Stockholm Sweden; ^4^ Icahn School of Medicine at Mount Sinai Department of Urology New York NY USA; ^5^ Karolinska Institutet Unit of Biostatistics Institute of Environmental Medicine (IMM) Stockholm Sweden; ^6^ Charité – University Hospital Medical Department Division of Nephrology Berlin Germany; ^7^ University College London Hospital London England

**Keywords:** education, prostate, robotic‐assisted radical prostatectomy, simulator, training, validation, virtual reality

## Abstract

**Background:**

Full‐procedure virtual reality (VR) simulator training in robotic‐assisted radical prostatectomy (RARP) is a new tool in surgical education.

**Methods:**

Description of the development of a VR RARP simulation model, (RobotiX‐Mentor®) including non‐guided bladder neck (ngBND) and neurovascular bundle dissection (ngNVBD) modules, and assessment of face, content, and construct validation of the ngBND and ngNVBD modules by robotic surgeons with different experience levels.

**Results:**

Simulator and ngBND/ngNVBD modules were rated highly by all surgeons for realism and usability as training tool. In the ngBND‐task construct, validation was not achieved in task‐specific performance metrics. In the ngNVBD, task‐specific performance of the expert/intermediately experienced surgeons was significantly better than that of novices.

**Conclusions:**

We proved face and content validity of simulator and both modules, and construct validity for generic metrics of the ngBND module and for generic and task‐specific metrics of the ngNVBD module.

## 1 INTRODUCTION

The development of structured and validated training curricula is one of the current priorities in robotic‐assisted urological surgery. The European Association of Urology (EAU) robotic training curriculum is a validated structured program to provide standardized training and to certify surgeons for specific urologic procedures.^1^ Hands‐on virtual reality (VR) simulator training is a major component in the early EAU robotic training curriculum using VR modules, based on the Fundamentals of Robotic Surgery (FRS).^1^, ^2^ A number of VR robotic simulators is commercially available. The simulator that we used for this study, the RobotiX Mentor® (3D Systems; Simbionix Products, Cleveland, OH, USA), is a robotic surgery VR simulator that has been developed to train surgeons using the da Vinci® Surgical System (Intuitive Surgical, Sunnyvale, CA, USA). The simulator platform consists of a height adjustable headset containing stereoscopic visors, free‐floating hand controls, and adjustable foot pedals integrated into a single console. It has been proven by Whitaker et al.^3^ to be effective for training using the FRS curriculum, a basic VR module. Currently, very few full procedure VR simulations exist to train trainees in performing all aspects of a robotic procedure. Development of non‐guided procedural training is likely to become an integral and essential part of modern urological robotic training curricula. Developments in VR techniques and affordable hardware have led to the production of full procedural VR simulations. Full procedural VR simulations have the potential to train surgeons in advanced technical, nontechnical, and cognitive skills, as well as in operation‐specific surgical steps that cannot be realized in basic VR modules. Before implementing full procedural VR modules into a robotic urological training curriculum, face, content, and construct validity of each step within a module need to be investigated and evaluated. Recently, Harrison et al.^4^ could proof construct validity of the RobotiX Mentor®'s guided bladder neck dissection (BND) and the urethrovesical anastomosis (UVA) step of the robotic‐assisted radical prostatectomy (RARP) module.

The aim of this prospective study was to describe the development of the non‐guided bladder neck dissection (ngBND) and non‐guided neurovascular bundle dissection (ngNVBD) in a VR simulation model (RobotiX Mentor®), and to perform the first assessment of face, content, and construct validation of the ngBND and ngNVBD in the full procedural RARP training module of the RobotiX Mentor® robotic surgery VR simulator.

## 2 MATERIALS AND METHODS

### 2.1 Study design

The project was carried out in two phases: (1) the design and development of the VR RARP simulation and (2) its evaluation and validation. In the development phase, multiple high definition (HD) videos of RARP, carried out by one expert high‐volume surgeon from Karolinska University Hospital, Stockholm, Sweden, were recorded. The procedure was divided into phases, one of which included the bladder neck dissection (BND) and one the neurovascular bundle dissection (NVBD). Using task deconstruction, each phase was broken down into defined tasks to be completed and the associated anatomical landmarks. Then, the tasks were defined in an objective ‘binary’ manner, and within each task, important visual cues, surgical errors, and events to be avoided were identified. The VR simulation was then built, taking into account these defined metrics. Table 1 describes the important visual cues to be identified in the VR simulation, as well as errors and events identified as important metrics for surgical performance. These metrics were integrated into the automated scoring system of the RobotiX Mentor® that rates the surgeon's performance.

**TABLE 1:** Important metrics identified to define surgical performance during VR RARP simulation

Module (phase)	Visual cues	Errors	Events
Bladder neck dissection (with BN preservation)	1. Define the prostate between instruments	1. Incorrect starting place for incision	1. Bladder wall undermined
	2. Bladder pedicles	2. Too cranial on the bladder	2. Button hole in the bladder
	3. Surgeon uses bladder stretch	3. Too close to the prostate	3. Damage to the UO's
	4. Area of fat lateral between bladder, prostate and NVB	4. Too much bleeding that obstructs view of surgical plane	4. Cut into the prostate
	5. Longitudinal muscle fibers of urethra	5. Inappropriate handling/trauma to the BN	5. Damage to the ureters/UO's
	6. UO's identified inside bladder neck		
Preparation and dissection of the pedicles and NVBD	1. Prostate	1. Inappropriate handling or excessive traction to NVB	1. Cut into prostate
	2. Denonvillier's fascia (post)	2. Incision of Denonvillier's fascia in the incorrect plane or direction	2. Cut across NVB
	3. Prostatic pedicle	3. Not mobilizing the prostate with the assistant arm to view dissection plane	3. Clips placed across the NVB
	4. Peri‐prostatic fascias		
	5. Prostatic capsule		
	6. Ability to visualize NVB to perform NVBD		
	7. Ability to visualize the prostate capsule and the ‘whiter’ prostate tissue if the capsule is breached		
	8. Dissection of the NVB of the apex of the prostate (completing the dissection)		

Abbreviations: BN, bladder neck; NVB, neurovascular bundle; NVBD, neurovascular bundle dissection; RARP, robotic‐assisted radical prostatectomy; UO, ureteral orificium; VR, virtual reality.

In the next phase, we aimed to evaluate both the RobotiX Mentor® console and its modules of ngBND and ngNVBD as parts of the full procedural VR RARP training module. This evaluation and validation was performed as a prospective, observational, and comparative study.

### 2.2 Participants

Subjects were categorised into three groups (novices, intermediates, and experts). Categorisation was based on the number of procedures required to reach proficiency in RARP.

Experts were individuals who had performed at least 100 RARP independently, were experienced as trainers, and who were presently and regularly performing RARP.^5^ The intermediately experienced group included residents and fellows receiving surgical training who had performed less than 100 cases. The novice group consisted of individuals who had no previous console operative experience, but may have had tableside assistance experience. Participants were recruited at Karolinska University Hospital and Medical School, Stockhom, Sweden, and at the 13th edition of the meeting of the EAU Robotic Urology Section (ERUS).

### 2.3 Power analysis

The power analysis was calculated with a two‐tailed test, with *α* = 0.05 and power (1 − β) = 0.80, and an intended reduction of 30% in time taken to complete each of the various tasks and stages for highly experienced robotic surgeons (more than 500 independent RARP cases), versus moderately experienced surgeons (20–50 RARP cases), versus robotic naïve medial students, based on data from previous studies.^6^, ^7^, ^8^ This calculation revealed that 8 subjects per group would be sufficient for finding statistically significant differences, which we increased to at least 15 per group to allow for the possible occurrence of dropouts and technical malfunctions of the simulator, and to compensate the less restrictive definition of expert level with at least 100 prostatectomies performed.

### 2.4 Curriculum

- Informed consent.
- Basic skills training (one time each module to become familiar with the simulator) two modules:
  - “Robotic Basic Skills Task 4 – Wristed Manipulation Level 2” (“Robotic Basic Skills Task 1 ‐ Camera 0”) (Module “Robotic Basic Skills Task 1 – Camera 0” is optional for the novice group in case struggling with the module “Robotic Basic Skills Task 4 – Wristed Manipulation Level 2” appears).
  - “Fundamentals of Robotic Surgery – Vessel Energy Dissection.”
- Non‐guided BND (full bladder‐neck sparing) for study purposes (one time).
- Non‐guided NVBD (full nerve‐sparing) for study purposes, both sides, start with right side (one time).
- Validation questionnaire.
- Evaluation questionnaire.

The study protocol was in compliance with the Declaration of Helsinki and in accordance with the regulations of the local Ethics Committee of the Karolinska Institutet, which approved the study; it was discussed and decided at the local institutional ethical review board that no formal ethical assessment is required as it does not involve patients. However, all participants were given written information on the study and asked to sign a participation consent form. All data collected was anonymised with no individual reports.

**FIGURE 1:**
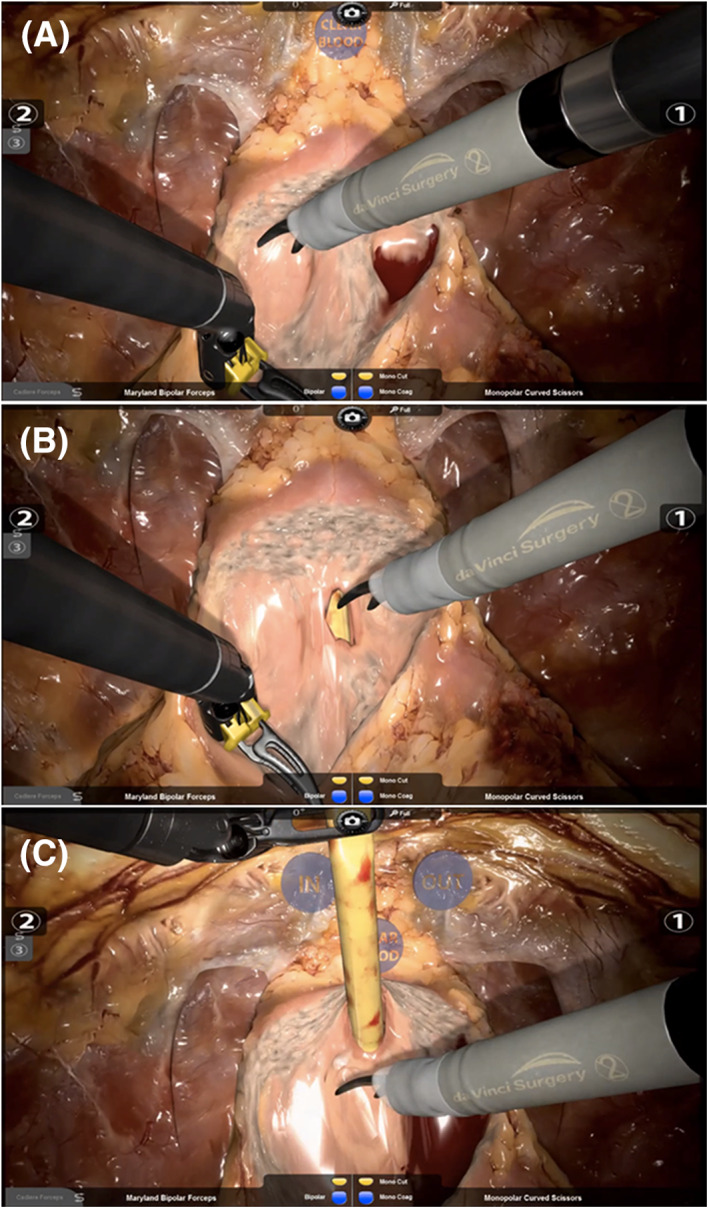
Scenes of the non‐guided full bladder‐neck sparing bladder neck dissection (ngBND) task. A, Showing the bladder neck completely freed. The icon “CLEAR BLOOD” provides a virtual sucker. Opening and closing the right‐handed instrument 1 or left‐handed instrument 2 on the icon activates a functional icon. B, Showing the ventral part of the urethra opened by the curved scissors close to the prostate for maximum bladder neck preservation. The yellow transurethral catheter becomes visible. C, Showing the ventral and dorsal parts of the bladder neck opened in a proceeded phase of the ngBND module. The third robotic instrument arm (fourth arm) is pulling up the transurethral catheter towards the symphysis. The virtual icons “IN” and “OUT” can pull the free catheter in or out

**FIGURE 2:**
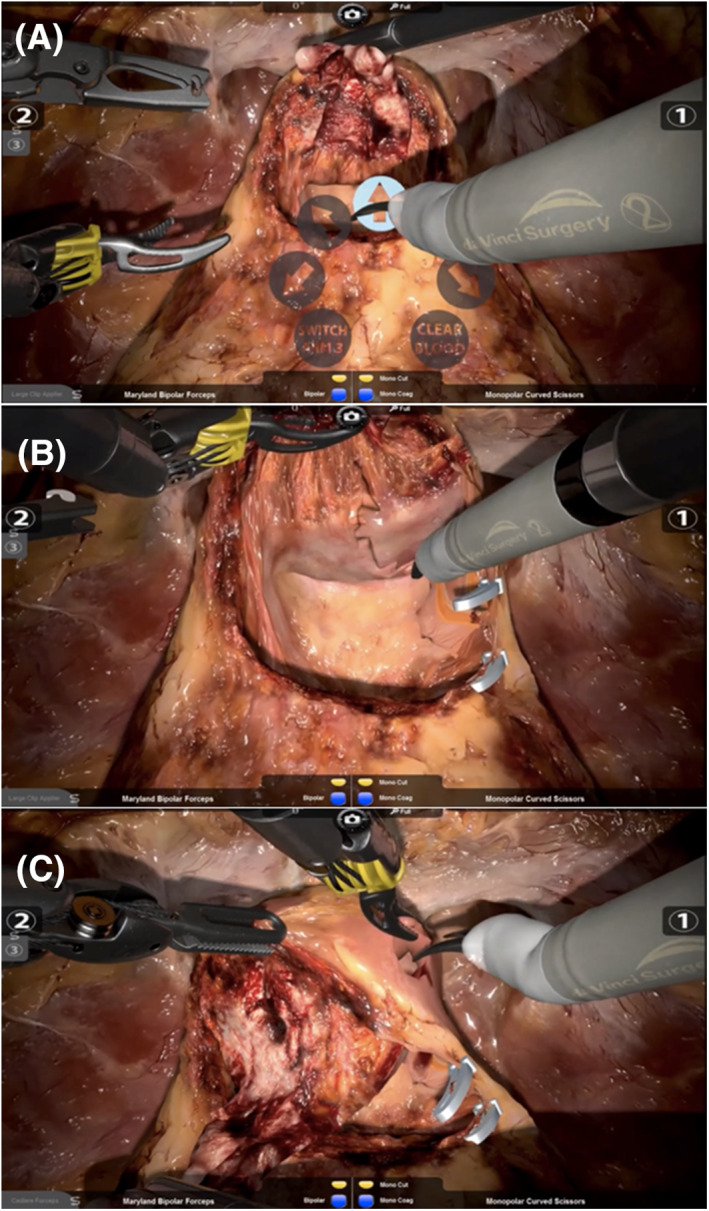
Scenes of the non‐guided full nerve–sparing neurovascular bundle dissection (ngNVBD) module. A, Showing the console surgeon using the virtual icon wheel to control the laparoscopic assistant arm, which is subsequently pulling up the seminal vesicles towards the camera. This functional icon wheel also controls the sucker (“CLEAR BLOOD”) and instrument change of the third instrument arm (“SWITCH ARM 3”: provides forceps and large‐medium Hemolock® clip applier). B, Showing a proceeded phase of the ngNVBD module. The right bladder pedicle was controlled by Hemolock® clips and dissected. The right neurovascular bundle was partially released in the dorsal parts. C, Showing a further proceeded phase of the ngNVBD module. The right neurovascular bundle (NVB) was partially released in the dorsal parts. The right NVB is dissected by high anterior‐lateral release

All novice surgeons initially underwent basic skills training on the RobotiX Mentor® console, which consisted of guiding the novices through the controls and teaching basic robotic skills. Intermediates and experts were offered the opportunity to complete a “familiarisation” task prior to commencing the procedural study modules. No data was collected from the familiarisation tasks. All participants then performed the ngBND task, aiming for full bladder neck‐sparing (Figure 1(A–C)). The participants were asked to accurately find the right plane between prostate and bladder before starting with the ngBND; they should clear the lateral periurethral parts (Figure 1(A)) before opening the urethra (Figure 1(B)), and were told to control the task for bleeding by using the suction mode and bipolar coagulation. Monopolar energy was not provided for the scissors. A fourth arm was provided to control the catheter with a forceps (Figure 1(C)). The ngBND task was followed by the ngNVBD task, aiming for full nerve‐sparing (Figure 2(A–C)), starting with the right side; the participants should aim for interfascial maximum nerve sparing with high anterior‐lateral release of the NVB using the scissors (Figure 2(C)) following control of the bladder pedicles with Hemolock® clips (Figure 2(B)). Dissection of the NVB was also performed by using Hemolock® clips. The option of metallic clips was not available. The ngNVBD module of the RobotiX Mentor® simulator uses a virtual laparoscopic assistant arm that is currently guided through a functional icon wheel controlled by the console surgeon. This icon wheel also controls the sucker and instrument change of the fourth arm (forceps and large‐medium Hemolock® clip applier; Figure 2(A)). The left‐handed arm was equipped with a Maryland bipolar forceps in both ngBND and ngNVBD. The performance measures in ngBND and ngNVBD of the participants was objectively recorded by the automated scoring system (MentorLearn) of the RobotiX Mentor®.

Post‐completion, all participants were asked to complete an evaluation questionnaire assessing their experience and opinion on realism of the simulator and of the ngBND and ngNVBD task, as compared to the da Vinci robotic system (face validity). Furthermore, the participants were asked to complete a validation questionnaire assessing the importance of robotic simulator training in general, and the importance, acceptability, and feasibility of the modules (content validity). Both questionnaires were developed by the King's College London as previously published,^3^, ^4^ and modified for our study purposes. The questionnaires are provided as supporting information (evaluation questionnaire and validation questionnaire). A 5‐point Likert scale was used for scoring (1 = worst ranking, 5 = best ranking). All data are presented as median with interquartile range (IQR).

## 3 RESULTS

A total of 51 participants completed the study; their demographics are shown in Table 2.

**TABLE 2:** Participant demographics

	Overall	Experts	Intermediates	Novices	*p*‐Value
No. of participants	51	18	16	17	‐
Age in years, median (IQR)	38.5 (31.0–45.0)	48.0 (43.0–51.0)	39.0 (35.5–40)	25.0 (22.5–34)	<0.001
Male gender, *n* (%)	47 (92.2)	17 (94.4)	14 (87.5)	16 (94.1)	0.68
Experience: median (IQR) number of robotically completed					
Prostatectomies observed	‐	‐	‐	1.5 (1.0–4.5)	‐
Cystectomies observed	‐	‐	‐	0.0 (0.0–1.0)	‐
Nephrectomies observed	‐	‐	‐	0.0 (0.0–0.0)	‐
Prostatectomies assisted	‐	‐	110.0 (140.0–300.0)	0.0 (0.0–1.0)	‐
Cystectomies assisted	‐	‐	10.5 (5.0–50.0)	0.0 (0.0–0.0)	‐
Nephrectomies assisted	‐	‐	10.0 (1.0–30.0)	0.0 (0.0–0.0)	‐
Prostatectomies performed	‐	500.0 (275.0–850)	12.5 (5.0–80.0)	‐	‐
Cystectomies performed	‐	15.0 (0.0–87.5)	0.0 (0.0–0.0)	‐	‐
Nephrectomies performed	‐	80.0 (0.0–250.0)	0.0 (0.0–2.0)	‐	‐
Technical skills training					
Formal training received, *n* (%)	24 (47.1)	10 (55.6)	14 (87.5)	0 (0)	<0.001
Assessment received, *n* (%)	17 (33.3)	7 (38.9)	9 (56.3)	1 (5.9)	0.007
Simulation experience, *n* (%)	29 (56.9)	12 (66.7)	13 (81.3)	4 (23.5)	0.003
Simulation courses attended, *n* (%)	13 (25.5)	5 (27.8)	7 (43.8)	1 (5.9)	0.04

### 3.1 Evaluation of the ngBND task performance:

In general, the intermediately experienced surgeons performed the task most accurately, followed by the experts and the novices, but these differences were not statistically significant (97.5 [91.5–99.7] vs. 94.7 [81.2–99.5] vs. 92.2 [83.4–99.4], *p* = 0.76)

The number of movements of the right, left, and fourth instruments decreased significantly with increasing degrees of expertise, and was therefore lowest in the expert group. Additionally, the total moving distance of the right and left instruments became significantly smaller with increasing expertise. Experts caused 3.6‐times and intermediates 2.1‐times less instrument collisions compared to novices (*p* < 0.01). Furthermore, the total time during which the instruments were used out of sight decreased with the level of expertise (*p* < 0.01). The total time to complete the ngBND module was longest in the intermediate group and shortest in the novice group (*p* < 0.01). Results of the ngBND task performance of all three groups are summarized in Table 3.

**TABLE 3:** Evaluation of candidates' performance in the non‐guided Bladder‐Neck‐Dissection (ngBND) task using the RobotiX Mentor simulator

Variable	Overall	Experts	Intermediates	Novices	*p*‐Value
Accuracy in %, median (IQR)	95.1 (83.4–99.6)	94.7 (81.2–99.5)	97.5 (91.5–99.7)	92.2 (83.4–99.4)	0.76
Bladder neck sides cleared prior to further dissection, *n* (%)	32 (62.7)	15 (75.0)	6 (42.9)	11 (64.7)	0.42
Bladder neck transection, *n* (%)	49 (96.1)	18 (100.0)	14 (87.5)	17 (100.0)	0.09
Catheter drops, median (IQR)	0 (0–1)	0 (0–1)	0 (0–1)	1 (0–1)	0.56
Correct catheter positioning during posterior dissection in %, median (IQR)	0.0 (0.0–33.2)	0.0 (0.0–60.2)	0.0 (0.0–78.7)	0.0 (0.0–0.0)	0.16
Clutch usage, median (IQR)	8 (5–11)	7.5 (4–15)	8.5 (6–11.5)	6 (4–9)	0.36
Complete haemostasis, *n* (%)	30 (58.8)	9 (50.0)	8 (50.0)	13 (76.5)	0.19
Dissection into bladder, median (IQR)	4 (2–21)	7.5 (2–21)	3 (1.5–9)	7 (1–21)	0.46
Dissection into prostate, median (IQR)	0 (0–1)	0 (0–1)	0 (0–1)	1 (0–3)	0.17
Distance by camera in mm, median (IQR)	784.8 (583.6–1068.7)	722.1 (591.5–981.1)	957.8 (611.0–1212.3)	784.8 (499.2–1010.7)	0.38
Injury of secondary structures, median (IQR)	1 (0–3)	1 (0–3)	0 (0–2)	1 (0–2)	0.29
Instrument collision, median (IQR)	30 (16–61)	17 (11–27)	28.5 (18.5–46)	61 (32–82)	<0.01
Movements of fourth instrument, median (IQR)	60 (37–82)	25.7 (17–58)	31.1 (40–82)	73 (58–89)	<0.01
Movements of left instrument, median (IQR)	503 (361–686)	354.5 (255–444)	525 (424.5–657)	686 (606–858)	<0.01
Movements of right instrument, median (IQR)	638 (258–1347)	415 (342–488)	644 (516.5–781)	746 (670–901)	<0.01
Distance travelled by fourth instrument in cm, median (IQR)	66.2 (40.3–96.3)	61.8 (19.3–80.0)	51.5 (35.3–92.7)	89.3 (60.6–109.1)	0.06
Distance travelled by left instrument in cm, median (IQR)	409.4 (320.1–600.8)	314.7 (206.5–379.9)	433.8 (325.4–524.9)	600.8 (451.8–698.3)	<0.01
Distance travelled by right instrument in cm, median (IQR)	474.7 (351.0–633.7)	341.5 (256.8–474.7)	472.5 (433.7–569.3)	559.5 (497.7–694.3)	<0.01
Occasions instruments are out of sight, median (IQR)	29 (20–53)	32.5 (21–49)	37 (20.5–77)	26 (19–40)	0.66
Total distance instruments are out of sight in cm, median (IQR)	22.4 (10.7–47.3)	22.6 (13.2–31.9)	15.4 (9.5–48.9)	22.4 (7.7–47.3)	0.88
Total time instruments are out of sight in seconds, median (IQR)	693 (431–863)	422 (289–545)	753.5 (542.5–861)	856 (714–1106)	<0.01
Total time in seconds, median (IQR)	728 (469–1130)	443.5 (383–636)	751 (611.5–920)	402.7 (1031–1472)	<0.01

### 3.2 Evaluation of the NVBD task performance:

The numbers of movements of the right, left, and fourth instrument were lowest in the expert group; statistically significant differences were found for the movements of the right instrument (*p* = 0.01) and fourth instrument (*p* = 0.02). The total moving distance of each instrument arm was smallest in the expert group, but the difference between the groups was statistically significant only for the fourth arm (*p* = 0.045). In addition, no significant differences were recorded for the number of instrument collisions. The total distance travelled by camera was smallest in the novice group and largest in the intermediate group (*p* < 0.01). Novices had less often occasions where instruments were out of sight compared to intermediates and also experts (*p* < 0.01). However, the total time during which instruments were out of sight significantly increased from the expert to the novice group (*p* = 0.03).

Concerning the nerve‐sparing prostatectomy procedure, the proportion of nerve sparing on the right side was statistically significantly worse in the novice group (72.8 [61.6–95.9]%) compared to the intermediate (98.7 [76.9–99.6]%) and the expert group (96.6 [83.5–98.4]), with experts showing the smallest IQR (*p* = 0.04). Nerve sparing on the left side was still worse in the novice group than in the intermediates and the experts, but due to a median 13.1% increase in the amount of nerve sparing on the left side compared to the right side in the novice group, this difference was no longer statistically significant. Clearly, suspected damage to the NVB was two‐times higher in the novice group compared to both other groups (*p* = 0.04). However, primary control of the vascular pedicles was rarely performed in all three groups, but was least likely carried out in the expert group (16.7%), *p* = 0.26. The total time to perform the ngNVBD task significantly decreased with increased level of expertise (*p* < 0.01). Further metrics are displayed in Table 4.

**TABLE 4:** Evaluation of candidates' performance in the non‐guided Neurovascular‐Bundle‐Dissection (ngNVBD) task using the RobotiX Mentor simulator

	Overall	Experts	Intermediates	Novices	*p*‐Value
Clutch usage, median (IQR)	11 (6–16)	9.5 (6–16)	14 (8–16)	6 (4–14.5)	0.17
Tissue injury with potential PSM, median (IQR)	4 (2–9)	6.5 (2–10)	4 (2–9)	4 (1–8.5)	0.46
Distance travelled by camera in cm, median (IQR)	179.4 (129.5–233.2)	186.4 (131.5–248.2)	214.6 (176.8–322.9)	129.6 (93.6–183.6)	<0.01
Injury to vascular pedicle, median (IQR)	1 (0–3)	1 (1–2)	1 (0–3)	1.5 (0–3)	0.91
Instrument collisions, median (IQR)	46 (35–72)	43 (23–48)	72 (41–130)	43 (32–67)	0.10
Nerve sparing left in %, median (IQR	91.8 (78.3–99.4)	99.1 (77.5–99.6)	91.6 (78.9–98.7)	85.9 (75.6–94.2)	0.23
Nerve sparing right in %, median (IQR)	95.6 (70.6–98.7)	96.6 (83.5–98.4)	98.7 (76.9–99.6)	72.8 (61.6–95.9)	0.04
Poor clip handling, median (IQR)	1 (0–2)	1 (0–2)	1 (0–2)	1.5 (0–2.5)	0.78
Primary control of vascular pedicles, *n* (%)	14 (27.5)	3 (16.7)	4 (24.0)	7 (41.2)	0.26
Respect for tissue, median (IQR)	12 (8–18)	11.5 (8–18)	12 (8–16)	14 (7–23)	0.69
Suspected damage to NVB, median (IQR)	5 (3–8)	4.5 (3–6)	5 (2–6)	9 (4–11)	0.04
Suspected injury to rectum, median (IQR)	0 (0–0)	0 (0–0)	0 (0–0)	0 (0–0)	0.81
Movements of fourth instrument, median (IQR)	151 (100–233)	107.5 (76–151)	205 (106–275)	168.5 (126–328.5)	0.02
Movements of left instrument, median (IQR)	410 (296–671)	339.5 (266–487)	493 (315–703)	572.5 (224.5–912.5)	0.15
Movements of right instrument, median (IQR)	877 (680–1122)	712.5 (611–876)	1122 (762–1429)	897 (752.5–1063.5)	0.01
Distance travelled by fourth instrument in cm, median (IQR)	275.0 (181.8–465.3)	199.3 (135.3–361.6)	359.3 (226.8–492.5)	275.0 (235.1–619.9)	0.046
Distance travelled by left instrument in cm, median (IQR)	352.6 (248.8–524.7)	297.4 (204.9–407.9)	386.5 (271.1–586.1)	420.1 (156.5–714.1)	0.26
Distance travelled by right instrument in cm, median (IQR)	671.2 (515.0–901.4)	635.0 (465.0–799.3)	895.4 (644.7–1017.4)	657.6 (539.8–886.6)	0.09
Occasions instruments are out of sight, median (IQR)	54 (31–76)	59 (35–74)	76 (52–104)	31.5 (21–54)	<0.01
Total distance instruments are out of sight in cm, median (IQR)	187.3 (132.1–349.8)	166.2 (98.3–263.2)	239.6 (161.6–414.0)	219.0 (140.0–375.4)	0.31
Total time instruments are out of sight in seconds, median (IQR)	683 (520–956)	589 (436–683)	802 (577–1218)	859 (450.5–1234)	0.03
Total time in seconds, median (IQR)	989 (762–1352)	757 (634–920)	1258 (878–1409)	1352.5 (1073.5–1571.5)	<0.01

### 3.3 Evaluation of realism of the RobotiX Mentor® simulator and modules

Overall, participants in all three subgroups assessed the realism of the RobotiX Mentor simulator, the ngBND, and the ngNVBD task as comparable to the da Vinci® Robot.

The overall ratings and the subgroups' ratings for the RobotiX Mentor® simulator and both modules are presented in detail in Table 5.

**TABLE 5:** (A) Evaluation of realism of the RobotiX Mentor® simulator; (B) realism of the non‐guided bladder neck dissection (ngBND) task; and (C) non‐guided neurovascular bundle dissection (ngNVBD) task compared to the da Vinci® surgical Robot

	Overall	Experts	Intermediates	Novices	*p*‐Value
(A) Realism of RobotiX Mentor simulator					
Overall experience, median (IQR)	3 (3–4)	3 (3–4)	3 (3–4)	4 (4–4)	0.07
Hand controls, median (IQR)	4 (3–4)	3 (3–4)	4 (3–4)	4 (3–4.5)	0.65
Graphics, median (IQR)	3 (3–4)	3 (3–4)	4 (2–4.5)	3.5 (3–4)	0.71
Clutch pedal, median (IQR)	5 (4–5)	5 (4–5)	4 (3–5)	5 (4–5)	0.60
Camera pedal, median (IQR)	5 (4–5)	5 (4–5)	4.5 (4–5)	5 (4–5)	0.75
Instrument swap pedal, median (IQR)	5 (4–5)	5 (4–5)	4 (4–5)	5 (3.5–5)	0.65
Diathermy pedals, median (IQR)	5 (4–5)	5 (4–5)	5 (4–5)	5 (3.5–5)	0.96
(B) Realism of ngBND task					
Cutting of tissues, median (IQR)	3 (2–4)	3 (2–4)	3 (2–4)	4 (3–4)	0.16
Bleeding/coagulation of vessels, median (IQR)	3 (2–4)	3 (2–3)	3 (2–3.5)	4 (3–4)	0.02
Clipping of tissues, median (IQR)	3 (3–4)	3 (3–4)	3 (3–4)	4 (3–5)	0.24
Behavior of tissues, median (IQR)	3 (2–4)	3 (2–3)	3 (2–4)	3.5 (3–4)	0.06
Realism of anatomy, median (IQR)	4 (3–5)	3 (3–4)	4 (3–5)	4 (4–5)	0.06
Camera movement, median (IQR)	5 (4–5)	5 (4–5)	4.5 (4–5)	5 (4–5)	0.80
Behavior of instruments, median (IQR)	4 (3–5)	4 (3–4)	4 (3–5)	5 (4–5)	0.22
Change of instruments, median (IQR)	4 (3–5)	3 (3–4)	4 (3–5)	4 (4–5)	0.11
Using third instrument, median (IQR)	4 (3–5)	3 (3–4)	4 (3–5)	4 (4–5)	0.25
(C) Realism of ngNVBD task					
Cutting of tissues, median (IQR)	3 (2–4)	3 (2–4)	3 (2–4)	4 (3–4)	0.34
Bleeding/coagulation of vessels, median (IQR)	3 (2–4)	3 (2–4)	3 (2–4)	4 (3–4)	0.05
Clipping of tissues, median (IQR)	4 (3–4)	3 (3–4)	4 (2.5–4.5)	4 (3–5)	0.22
Behaviour of tissues, median (IQR)	3 (3–4)	3 (2–3)	3 (2.5–4)	4 (3–4)	0.16
Realism of anatomy, median (IQR)	4 (3–5)	4 (3–4)	4 (3–4.5)	5 (4–5)	0.10
Camera movement, median (IQR)	4 (4–5)	4 (3–5)	4 (3.5–5)	5 (4–5)	0.58
Behaviour of instruments, median (IQR)	4 (4–5)	4 (4–5)	4 (4–5)	5 (4–5)	0.52
Change of instruments, median (IQR)	4 (3–5)	4 (3–4.5)	4 (3–5)	5 (4–5)	0.33
Using third instrument retraction, median (IQR)	4 (3–4)	4 (3–4)	4 (3–4)	4 (4–5)	0.58
Using third instrument clipper, median (IQR)	4 (3–4)	4 (3–4)	3 (3–4)	4 (3–5)	0.23
Using irrigation, median (IQR)	3 (2.5–4)	3 (3–4)	3 (2–4)	4 (3–4)	0.51
Using virtual assistant, median (IQR)	3 (3–4)	4 (2–4)	3 (3–4)	3 (3–4)	1.00

*Note:* 1 = strongly disagree/not similar, 5 = strongly agree/very similar.

The ratings for realism of the ngBND task were significantly different among the three participating groups concerning: bleeding/coagulation of vessels, behaviour of tissues, and realism of anatomy, while in the ngNVBD task, significantly different results were only found for bleeding/coagulation of vessels, with novices tending to rate higher.

### 3.4 Validation of the RobotiX Mentor® simulator and console:

Overall, the acceptance rate of the RobotiX Mentor® simulator was high and it was rated as a valuable and feasible training tool by all three groups, but novices tended to award higher ratings in almost every category than expert and intermediate candidates.

Generally, the usefulness of both modules (ngBND and ngNVBD) was rated highly (4 [3–5] and 4 [4–5] points), but with only 3 (2–4) points, the experts rated the usefulness of the ngBND task lower than the ngNVBD task (4 [3–4]). Especially, the novices recognized an improvement in their robotic skills and confidence to perform a robotic surgery.

In addition, the usability of the console received high ratings from all participating groups.

However, experts appraised the use of the hand pedal and the diathermy pedal significantly worse than intermediates and novices. Also, the realism of the graphics was rated lower by the experts, but the difference was not statistically significant.

These results, along with the importance of several tasks for robotic surgical training, are shown in Table 6.

**TABLE 6:** (A) Validation of the RobotiX Mentor® as a training tool; (B) Validation of the RobotiX Mentor's console; and (C) Validation of the importance of special tasks for robotic surgical training

	Overall	Experts	Intermediates	Novices	*p*‐Value
(A) Validation of the RobotiX Mentor® as a training tool					
In your opinion, how useful did you find the BND module? Median (IQR)	4 (3–5)	3 (2–4)	4 (3–4)	5 (3.5–5)	0.01
In your opinion, how useful did you find the NVBD module? Median (IQR)	4 (4–5)	4 (3–4)	4 (4–4)	5 (4–5)	0.01
The RobotiX Mentor is a realistic training simulator for robotic surgery, median (IQR)	4 (3–4)	4 (3–4)	4 (3–4)	4 (3.5–5)	0.42
There is a role for the RobotiX Mentor in training for robotic surgery, median (IQR)	4 (4–5)	4 (4–4)	4 (4–5)	5 (5–5)	0.03
The RobotiX Mentor should be routinely used for training and assessment of robotic surgery, median (IQR)	4 (4–5)	4 (4–4)	4 (3–4)	5 (4.5–5)	0.002
The RobotiX Mentor is a good way to learn relevant robotic skills, median (IQR)	4 (4–5)	4 (4–4)	4 (3–4)	5 (5–5)	0.001
This session has improved my robotic skills, median (IQR)	3 (2–5)	2 (1–3)	3 (2–3)	5 (5–5)	<0.001
This session increased my confidence in performing robotic surgery, median (IQR)	3 (2–4)	2 (1–3)	2.5 (2–3)	3.5 (2.5–4.5)	0.08
I would recommend this to others, median (IQR)	4 (4–5)	4 (4–5)	4 (3–5)	5 (5–5)	0.03
How feasible is incorporating the RobotiX Mentor into a training programme? Median (IQR)	4 (3–5)	4 (3–5)	4 (3–5)	5 (4–5)	0.19
How acceptable is incorporating the RobotiX Mentor into the training programme? Median (IQR)	5 (4–5)	5 (4–5)	5 (4–5)	5 (4–5)	0.54
(B) Validation of the RobotiX Mentor's console					
In your opinion, how easy was it to use the hand controls? Median (IQR)	4 (3–4)	3 (3–4)	4 (3–4)	4 (4–5)	0.009
In your opinion, how realistic were the graphics? Median (IQR)	4 (3–5)	3 (3–4)	3 (3–5)	4 (3–5)	0.34
In your opinion, how easy was it to use the clutch pedal? Median (IQR)	5 (4–5)	5 (4–5)	4 (3–5)	5 (5–5)	0.06
In your opinion, how easy was it to use the camera pedal? Median (IQR)	5 (4–5)	5 (4–5)	5 (4–5)	5 (5–5)	0.27
In your opinion, how easy was it to use the instrument swap pedal? Median (IQR)	4 (3–5)	4 (3–5)	4 (3–5)	5 (4–5)	0.34
In your opinion, how easy was it to use the diathermy pedal? Median (IQR)	5 (4–5)	4 (4–5)	5 (4–5)	5 (5–5)	0.009
(C) In your opinion, how important are the following tasks for robotic surgical training:					
Tissue behavior in advanced simulation, median (IQR)	5 (3–5)	4 (4–5)	4 (3–5)	5 (4–5)	0.42
Tissue dissection/cutting, median (IQR)	4 (3–5)	4 (3–5)	4 (3–5)	4 (3–5)	0.67
Vessel dissection, median (IQR)	4 (3–5)	4 (3–5)	4 (3–5)	5 (4–5)	0.44
Vessel coagulation, median (IQR)	4 (3–5)	4 (3–4)	4 (3–5)	4 (4–5)	0.52
Clipping of tissue, median (IQR)	4 (3–5)	4 (3–4)	4 (3–5)	4 (3–5)	0.77
Realism of anatomy, median (IQR)	4.5 (4–5)	4 (4–5)	5 (4–5)	4 (4–5)	0.88
Camera movement, median (IQR)	5 (4–5)	4 (4–5)	5 (4–5)	5 (4–5)	0.20
Behavior of instruments, median (IQR)	5 (4–5)	4 (4–5)	4.5 (4–5)	5 (5–5)	0.07
Change of instruments, median (IQR)	4 (3–5)	4 (4–5)	4 (3–5)	4 (3–5)	0.94
Using 3^rd^ instrument retraction, median (IQR)	4 (3–5)	4 (3–5)	4 (3–5)	4 (3–5)	0.99
Using 3^rd^ instrument clipper, median (IQR)	4 (3–4.5)	4 (3–4)	4 (3–5)	4 (3–5)	0.60
Using irrigation, median (IQR)	4 (3–4)	3.5 (3–4)	4 (3–5)	4 (3–5)	0.57
Using virtual assistant, median (IQR)	4 (3–5)	4 (3–4)	4 (2.5–4.5)	4 (2–5)	1.00

*Note:* 1 = not useful/strongly disagree/not easy/not feasible/not acceptable/not important, 5 = very useful/strongly agree/very easy/very feasible/very acceptable/very important.

### 3.5 Validation of robotic simulator training:

The importance of robotic simulator training was mentioned by all three groups, with no significant difference. Of those having answered the question, all participants in each group agreed with the statement that simulation should be implemented into training programs.

However, only 68.8% of the participants affirmed that current VR simulation models should be part of accreditation/(re)certification, with no significant difference between the three groups. These results are depicted in Table 7.

**TABLE 7:** Validation of robotic simulator training

	Overall	Experts	Intermediates	Novices	*p*‐Value
Validation of robotic simulator training					
There is a role for a validated robotic simulation program in urology training, median (IQR)	5 (4–5)	5 (4–5)	4.5 (4–5)	5 (5–5)	0.17
Trainees should learn an operation on a simulator prior to operating on a live patient, median (IQR)	5 (4–5)	5 (4–5)	5 (4–5)	5 (5–5)	0.58
Simulation‐based training and assessment is essential for patient safety, median (IQR)	5 (4–5)	5 (4–5)	5 (4–5)	5 (4–5)	0.74
A full procedure simulation has a beneficial educational impact on surgical training, median (IQR)	5 (4–5)	5 (4–5)	5 (4–5)	5 (4.5–5)	0.82
Should simulation be implemented into training programs? yes, *n* (%)	48 (94.1)	17 (94.4)	16 (100.0)	15 (88.2)	0.15
Should simulation be part of accreditation/(re)certification?, yes, *n* (%)	35 (68.6)	14 (77.8)	9 (56.3)	12 (70.6)	0.23

*Note:* 1 = strongly disagree, 5 = strongly agree.

## 4 DISCUSSION

We performed the first face, content and construct validation of the ngBND and ngNVBD full procedural prostatectomy VR robotic training module of the RobotiX Mentor® robotic surgery VR simulator developed by Simbionix.

In general, the simulator and the ngBND and ngNVBD modules were rated highly by all three groups of different expertise in terms of their realism as well as their usefulness as a training tool. Similar to the participants of a study by Harrison et al.^4^ the participants in our study and in particular the experts tended to rate the realisms of the graphics and hand controls of the RobotiX Mentor® simulator worse than other metrics, which were all compared with the real da Vinci® Robot. Concerning the ngBND and ngNVBD module, intermediates and experts rated the bleeding and the coagulation of vessels in both tasks statistically significantly worse than the novices, possibly due to a higher level of experience along with better comparison options of more experienced surgeons. In addition, the anatomy of the ngNVBD module seemed to be more convincing to the experts than the ngBND module. This is also reflected by the slightly worse rating of the usefulness of the ngBND module by the expert group. However, the realism of the RobotiX Mentor® simulator and the ngBND and ngNVBD tasks is rated as comparable to the real da Vinci® robotic system when evaluated by the participants, which confirms face validity of the simulator. Consequently, the simulator is highly recommended as a routine training and assessment tool for robotic surgery by all participating groups. Nonetheless, the training effect that can be achieved by the simulator certainly reaches its limits. This is reflected by the finding that novices showed a stronger, and experts a less pronounced, improvement in their robotic skills, as compared to intermediates. This result supports face validity of the simulator system.

For the ngBND task, significant differences between the differently experienced groups was mainly seen in the generic automated performance metrics (APM) (e.g. less instrument collisions, less movements, and smaller moving distance by instruments with increased expertise), and also by the total time to finish the module, which decreased with the level of expertise, but not in task‐specific performance metrics. These findings are similar to APM assessments on the actual da Vinci® Robot.^9^ Interestingly, the total moving distance of the fourth arm was much smaller but was moved more often by the novices, representing an inefficient/more uncontrolled usage of the fourth arm in this group.

For the ngNVBD, short‐time learning effects following the ngBND module resulted in a trend towards levelling of the generic performance among the three groups in terms of number of instrument collisions and total moving distance of the instruments. However, the total moving distance of the fourth arm maintained statistically significantly different in the three groups and was lowest in the expert group, which indicates a generally more inefficient or more uncontrolled usage of the fourth arm in the other groups. An additional short‐time learning effect was shown in the novice group for the percentage of preservation of the NVB, which increased by 13.1% from the first nerve‐sparing side (right) to the second side (left), resulting in statistically significantly worse nerve‐sparing performance of the novices on the right but not on the left NVB, as compared to intermediates and experts. However, task‐specific performance was still better in intermediates and experts than in the novice group. The level of expertise is also reflected by the lower number of suspected damage to the NVB and the shorter time to finish the module in the more experienced groups. Another metric that might reflect expert status is the fact that experts showed nearly twice as often occasions with instruments out of sight than novices, but at the same time, the total time during which the instruments were out of sight was 31.4% shorter. This suggests that the experts' cognitive idea of instrument positioning and movement is deep‐seated and the simulator's transfer of the handgrip movements might not correspond to their individualized default settings in the da Vinci® system.

Recently, APMs, defined as instrument motion tracking metrics and synchronized surgical footage, captured with a novel data‐recording device, the “dVLogger” (Intuitive Surgical), directly from the da Vinci® robotic system in real time during the actual live surgical procedure, were investigated concerning their ability to assess surgical performance and patient outcomes. The data revealed that experts showed more efficient camera manipulation and smaller total distance between the two instruments (dominant and non‐dominant) in selected steps of RARP (BND and NVBD not investigated).^10^ Another study on robotic VR simulation exercises also suggested that expert surgeons had significantly less total camera moving time but higher frequency of camera movement than new robotic surgeons.^11^ Correspondingly, in our study, the total distance between the right and the left instrument was smallest in the expert group in both the ngBND task and ngNVBD task. The distance moved by camera did not differ significantly between the three groups in both tasks, it was smallest in the novice group and greatest in the intermediate group. However, it was previously shown that different steps of a RARP can cause different results regarding camera path length in expert and novice surgeons, with experts resulting in smaller camera path length in three out of four investigated steps.^10^

Thus, construct validity of the ngBND module was mainly proved for generic metrics, but not for task‐specific metrics. On the other hand, construct validity could be proven for the ngNVBD module in task‐specific metrics and, with less extend, for generic metrics, which were already biased by a short‐time learning effect in the less experienced groups, especially in the novices.

The realism of the cutting of tissue, bleeding/coagulation of vessels, clipping of tissue, and the behaviour of the tissue, was mainly scored with a median of three out of five points on the Likert scale in the intermediate and expert group. These results suggest that the simulator performance does not always meet the surgeons' requirements, especially in situations where tissue starts moving. As described for the urethrovesical anastomosis task,^4^ some participants described “floating clips” and described the tissue as “jelly‐like.” Consequently, problems with the setting of the clips to control the vascular pedicles in the NVBD module might have been the reason for the low rating of this metric in all three groups, but the lowest in the expert group. However, the realism of moving tissue and the realistic behaviour of tissue towards other objects, like clips, needles, or instruments, is demanding and represents a remarkably weak point with a potential for improvements in advanced and VR medical simulators, not only in the RobotiX Mentor® simulator. However, future products will allow for better processing, and new graphic cards will allow manufacturers to improve tissue behaviour.

A wide variety of procedures are conducted in urology, and thus a variety of simulation modalities have become available.^12^ Simulation‐based training is increasingly recognised as a valuable adjunct to training in urology and other disciplines. However, studies investigating the validity and usefulness of procedural VR simulations are rare and mainly limited to endourological and laparoscopic procedures.^13^ To our knowledge, 3D Systems Simbionix is the only company that provides complete robotic procedure simulated cases, which enables the performance of critical steps of various procedures, including RARP.^3^, ^4^ This structured method of training, called modular training, is supported by a good evidence base in urology,^14^ and numerous suggested training modules exist for minimally invasive procedures.^13^, ^14^ Modular RARP console and full‐procedure training has been established in the European Association of Urology Robotic Training Curriculum.^15^ This validated and structured modular console training allows surgeons to progressively perform surgical steps with increasing levels of complexity under supervision.^1^

In real live surgery, the recent development of a modular assessment tool for RARP allows for evaluation of learning curves for individual steps of the procedure, which provides mentors with an objective way to assess progression through the modules.^16^

In VR, full procedure modular training competency can be assessed by developing benchmark scores based on experts' performance.^17^ Consequently, the data of this study can be further used to establish a benchmark‐based modular training curriculum of full procedural radical prostatectomy modules. In addition, the data collected from the robot naïve medical student group will also provide further information on the variation in baseline “inherent” skills in VR robotic simulation. In future, utilizing machine learning and APMs to evaluate RARP performance in live surgery and predict patient outcomes^9^, ^18^ might influence the assessment of candidates performing VR full procedural RARPs.

However, the assumed additional benefit of VR full procedure radical prostatectomy modules in comparison to basic VR training needs to be further investigated.

The role of nontechnical skills in surgery is increasingly understood and represents an expanding area within the literature. It is now known that deficiencies in this area are a major source for surgical errors, and it is increasingly recognised that these skill sets are not peripheral but rather should be seen, alongside with technical skills, as being of core importance.^13^

The ngNVBD module of the RobotiX Mentor® simulator uses a virtual laparoscopic assistant arm, which is currently guided through a functional icon wheal by the console surgeon. The virtual assistant arm was highly rated in terms of realism, especially by the expert group. Nevertheless, we recommend implementing a voice control of the virtual assistant arm. This would provide the opportunity to also simulate a very important nontechnical skill, defined as “social skill,” including communication, teamwork, and leadership.^13^

Furthermore, the study participants agreed to implement simulation into training programs, whereas they disagreed on it being part of accreditation/(re)‐certification. This reflects the strong need to implement advanced VR full procedural prostatectomy modules, beside basic skills modules, into RARP surgical training curriculums, but at the same time raises the question of where to adequately place them to gain the most benefit in the surgical education and quality management process. The fact that some specific construct validity results, like the “achieved percentage of nerve sparing,” were already biased by a short‐time learning effect in the less experienced groups, especially in the novices, suggests that the simulation does currently not reflect the demanding real‐live learning curve to achieve good functional outcomes in RARPs.^9^, ^19^, ^20^ Therefore, we recommend joining the separate modules of a prostatectomy simulation to a complete surgical procedure, and designing opportunities which will allow creating new VR content very timely, including different challenging anatomical variations.

However, the results of our study and the validation need to be considered with caution, and further discussion should be directed to the methodology, the study process, and different stereotypical behaviours of groups with different levels of expertise. The outcome measurements are based on important metrics that were identified by means of assessments of videos displaying one expert RARP surgeon, and the identified metrics were then used to code and define surgical performance during VR simulation, which, in turn, are represented by the MentorLearn scores of the Simbionix simulator system. Therefore, different concepts of performing a BND or NVBD that lead to comparable good results in real surgery may have resulted in different metrics scored by the RobotiX Mentor system, especially in more experienced surgeons, and in particular when the tasks are performed non‐guided. For example, the unusual use of cold cuts and only bipolar coagulation mode in the ngBND task might have biased the task‐specific performances, missing to prove construct validity. Furthermore, skilful (brief, efficient, and frequent) camera manipulation is emerging as an important indicator of robotic surgical experience.^10^, ^11^, ^18^ Hence, future types of the Simbionix MentorLearn should follow certain APMs validated from live RARPs, and should also include the possibility to rate various other instrument performance metrics.

In our study, no guiding was applied during the tasks, but while performing the familiarisation tasks, all participants were informed about the relevant characteristics of the coded metrics in the study to prevent from coding‐related bias. However, the fact that, apart from the total performance time, no statistically significant differences in the ngBND task‐specific performances were found between the groups might be explained by this circumstance. On the other hand, Harrison et al.^4^ who validated the guided BND module of the RobotiX Mentor® simulator, had experienced problems to prove construct validity of the guided BND task, too. They argued that some intermediates and experts questioned the computer's technique for the task and were hesitant to perform the task as per the instructions provided by the simulator, whereas the novices followed the instructions readily.^4^

Experts and intermediates were defined as surgeons who had independently performed at least 100 or less than 100 RARP, respectively. Actually, the median (IQR) number of performed RARP was 500.0 (275.0–850.0) in the expert and 12.5 (5.0–80.0) in the intermediate group. This large difference might have influenced the results, but it also reflects the long learning curve that is necessary to achieve good oncological and functional results in RARP.^5^, ^9^, ^19^, ^20^ However, we performed the same analysis with expert level defined as 50 RARP, and found no relevantly different results.

In summary: novice, intermediate, and expert RARP surgeons evaluated the ngBND and ngNVBD full procedural VR training module of the Simbionix MentorX simulator and approved it as a realistic, feasible, and acceptable component for a robotic surgical training program. Construct validity was proved for generic performance metrics but not for task‐specific metrics in the ngBND module, and for both metrics in the ngNVBD module. Novices showed an increase in the percentage of nerve sparing between the right and the left side of the NVB, which indicates a significant learning effect.

Since validity has been described as a continuing argument,^21^ we believe our work makes an important contribution to the ongoing validation of the RobotiX‐Mentor® full‐procedure RARP VR simulation by describing important metrics identified to define surgical performance during VR RARP simulation, by analysing the performance of the study participants, and by comparing our results with previous validation results. However, validation is determined by evidence.^21^ Thus, further implementation of these modules into the curriculum and continued analysis would be beneficial to gauge its overall usability.

## CONFLICT OF INTEREST

The authors declare explicitly that they have no competing interests in connection with this article.

## AUTHOR CONTRIBUTIONS

J.E. designed the study, interpreted the data, and drafted the manuscript. J.W.C. designed and critically revised the manuscript for important intellectual content and interpreted the data. P.N.W., O.A., S.C., and M.J.O. were involved in data acquisition and interpretation, and revised the manuscript for important intellectual content. J.H. and M.H. were involved in the statistical analysis. All authors read, gave comments, and approved the final version of the manuscript.

## Supporting information

Supplementary Material
