# Supplementary material for: Development and validation of non‐guided bladder‐neck and neurovascular‐bundle dissection modules of the RobotiX‐Mentor® full‐procedure robotic‐assisted radical prostatectomy virtual reality simulation
Source: Int J Med Robot. 2020 Nov 13;17(2):e2195. doi: 10.1002/rcs.2195 (PMC7988553; doi:10.1002/rcs.2195)
Supplement: Supplementary file 1 — Supplementary Material [file RCS-17-e2195-s001.pdf]

Simbionix RobotiX Mentor Evaluation Questionnaire

-Your responses will be processed anonymously-

Section 1: General Demographics

Name: ………………………………………………………………………… Age: ………………..

Gender: M/F

Clinical Experience (please circle):

Trainee – stage of training:

CT1	CT2	ST3	ST4	ST5	ST6	ST7	ST8

Consultant – years of independent practice:

0-1	1-3	3-5	5-10	10-15	15-20	>20

Section 2: Previous Experience

Please indicate (**no. of times**) your experience for the following procedures:

Procedures	Observed	Assisted	Performed
**Robotic-assisted prostatectomy**			
**Robotic-assisted nephrectomy (inc. partial)**			
**Robotic-assisted cystectomy**			

Section 3: Technical Skills Training

1. Have you received formal training for robotic surgery? Y/N
2. Have you ever been assessed on your performance for robotic surgery? Y/N
3. Do you have simulation experience of robotic surgery? Y/N

4. Please indicate (tick) which methods of Simulation you previously used:

Robotic Surgical Simulator (RoSS)		Simsurgery Educational Platform (SEP)		ProMIS		Mimic dV-Trainer (mdVT)		Da Vinci Skills Simulator	

5. Have you attended any other robotic or simulation courses? If so, please specify

Y/N

…………………………………………………………………………………………

…………………………………………………………………………………………

Section 4: Simulator Evaluation (5=best rating, 1=worst rating)

1. Was your overall experience similar to that of the real da Vinci surgical robot?

**Not Similar** 1	2	3	4	5 **Very Similar**

2. Indicate your view on how realistic the **RobotiX Mentor** is in terms of:
   a. Hand controls 1☐ 2☐ 3☐ 4☐ 5☐
   b. Graphics 1☐ 2☐ 3☐ 4☐ 5☐
   c. Clutch pedal 1☐ 2☐ 3☐ 4☐ 5☐
   d. Camera pedal 1☐ 2☐ 3☐ 4☐ 5☐
   e. Instrument swap pedal 1☐ 2☐ 3☐ 4☐ 5☐
   f. Diathermy pedals 1☐ 2☐ 3☐ 4☐ 5☐

3. Indicate your view on how realistic the **BND** task is in terms of:
   a. cutting of tissues 1☐ 2☐ 3☐ 4☐ 5☐
   b. bleeding/coagulation of vessels 1☐ 2☐ 3☐ 4☐ 5☐
   c. Clipping of tissues 1☐ 2☐ 3☐ 4☐ 5☐
   d. Behavior of tissues 1☐ 2☐ 3☐ 4☐ 5☐
   e. Realism of anatomy 1☐ 2☐ 3☐ 4☐ 5☐
   f. Camera movement 1☐ 2☐ 3☐ 4☐ 5☐
   g. Behavior of instruments 1☐ 2☐ 3☐ 4☐ 5☐
   h. Change of instruments 1☐ 2☐ 3☐ 4☐ 5☐
   i. Using third instrument 1☐ 2☐ 3☐ 4☐ 5☐

4. Indicate your view on how realistic the **NVBD** tasks is in terms of:
   a. cutting of tissues 1☐ 2☐ 3☐ 4☐ 5☐
   b. bleeding/coagulation of vessels 1☐ 2☐ 3☐ 4☐ 5☐
   c. Clipping of tissues 1☐ 2☐ 3☐ 4☐ 5☐

d. Behavior of tissues 1☐ 2☐ 3☐ 4☐ 5☐
e. Realism of anatomy 1☐ 2☐ 3☐ 4☐ 5☐
f. Camera movement 1☐ 2☐ 3☐ 4☐ 5☐
g. Behavior of instruments 1☐ 2☐ 3☐ 4☐ 5☐
h. Change of instruments 1☐ 2☐ 3☐ 4☐ 5☐
i. Using 3rd instrument retraction 1☐ 2☐ 3☐ 4☐ 5☐
j. Using 3rd instrument clipper 1☐ 2☐ 3☐ 4☐ 5☐
k. Using irrigation 1☐ 2☐ 3☐ 4☐ 5☐
l. Using virtual assistant 1☐ 2☐ 3☐ 4☐ 5☐

(1=strongly disagree, 5=strongly agree)

5. The **RobotiX Mentor** is a realistic training simulator for robotic surgery: 1☐ 2☐ 3☐ 4☐ 5☐

6. There is a role for the **RobotiX Mentor** in training for robotic surgery: 1☐ 2☐ 3☐ 4☐ 5☐

7. The **RobotiX Mentor** should be routinely used for training and assessment of robotic surgery: 1☐ 2☐ 3☐ 4☐ 5☐

8. This session has improved my robotic skills: 1☐ 2☐ 3☐ 4☐ 5☐

9. The **RobotiX Mentor** is a good way to learn relevant robotic skills: 1☐ 2☐ 3☐ 4☐ 5☐

10. This session increased my confidence in performing robotic surgery: 1☐ 2☐ 3☐ 4☐ 5☐

11. I would recommend this to others: 1☐ 2☐ 3☐ 4☐ 5☐

12. There is a role for a validated robotic simulation programme in urology training: 1☐ 2☐ 3☐ 4☐ 5☐

13. Trainees should learn an operation on a simulator prior to operating on a live patient: 1☐ 2☐ 3☐ 4☐ 5☐

14. Simulation-based training and assessment is essential for patient safety: 1☐ 2☐ 3☐ 4☐ 5☐

15. A full procedure simulation has a beneficial educational impact on surgical training: 1☐ 2☐ 3☐ 4☐ 5☐

16. In your opinion, how important are the following tasks for robotic surgical training:

    a) tissue behavior in advanced simulation 1☐ 2☐ 3☐ 4☐ 5☐

b) tissue dissection/cutting 1☐ 2☐ 3☐ 4☐ 5☐
c) Vessel dissection 1☐ 2☐ 3☐ 4☐ 5☐
d) Vessel coagulation 1☐ 2☐ 3☐ 4☐ 5☐
e) Clipping of tissues 1☐ 2☐ 3☐ 4☐ 5☐
f) Realism of anatomy 1☐ 2☐ 3☐ 4☐ 5☐
g) Camera movement 1☐ 2☐ 3☐ 4☐ 5☐
h) Behavior of instruments 1☐ 2☐ 3☐ 4☐ 5☐
i) Change of instruments 1☐ 2☐ 3☐ 4☐ 5☐
j) Using 3rd instrument retraction 1☐ 2☐ 3☐ 4☐ 5☐
k) Using 3rd instrument clipper 1☐ 2☐ 3☐ 4☐ 5☐
l) Using irrigation 1☐ 2☐ 3☐ 4☐ 5☐
m) Using virtual assistant 1☐ 2☐ 3☐ 4☐ 5☐

17. Please rank the following six robotic surgical simulators:
(1=least recommended, 6=most recommended, if you have no experience of one simulator write NE beside that one)

**RobotiX Mentor (Simbionix)**		**Robotic Surgical Simulator (RoSS)**		**Simsurgery Educational Platform (SEP)**		**ProMIS**		**Mimic dV-Trainer (mdVT)**		**Da Vinci Skills Simulator**	

18. Should simulation be implemented into training programmes? Y/N

19. Should simulation be part of accreditation / (re)certification? Y/N

20. Do you think a full procedure simulation is beneficial for surgical education?

**Not Beneficial** 1	2	3	4	5	**Beneficial**

21. How feasible is incorporating the **RobotiX Mentor** into the training programme?

**Not Feasible** 1	2	3	4	5	**Feasible**

22. How acceptable is incorporating the **RobotiX Mentor** into the training programme?

**Not Acceptable** 1	2	3	4	5	**Acceptable**

Thank you for your kind participation!

KING'S College LONDON

Karolinska Institutet

Simbionix RobotiX Mentor Validation Questionnaire

-Your responses will be processed anonymously-

Section 1: Demographics

Name: ……………………………........

Age: ……………………………….....

Year of study: ………………………

Section 2: Advanced simulation: full procedure RARP

Please answer the following questions regarding the advanced simulation surgery for BND and NVBD. (5=best rating, 1=worst rating)

1. In your opinion, how useful did you find the modules?

1☐ 2☐ 3☐ 4☐ 5☐

Section 3: Robotic BND

Please answer the following questions regarding the BND module.

2. In your opinion, how useful did you find the BND module?

1☐ 2☐ 3☐ 4☐ 5☐

3. What further additions could be made to this module?

……………………………………………………………………………………

……………………………………………………………………………………

……………………………………………………………………………………

Section 4: Robotic NVBD

Please answer the following questions regarding the NVBD module.

4. In your opinion, how useful did you find the NVBD module?

1☐ 2☐ 3☐ 4☐ 5☐

5. What further additions could be made to this module?

………………………………………………………………………………………………

………………………………………………………………………………………………

………………………………………………………………………………………………

Section 5: Features of RobotiX Mentor

Please answer the following questions regarding the RobotiX Mentor console. (5=best rating, 1=worst rating)

6. In your opinion, how easy was it to use the hand controls?

   1☐ 2☐ 3☐ 4☐ 5☐

7. In your opinion, how realistic were the graphics?

   1☐ 2☐ 3☐ 4☐ 5☐

8. In your opinion, how easy was it to use the clutch pedal?

   1☐ 2☐ 3☐ 4☐ 5☐

9. In your opinion, how easy was it to use the camera pedal?

   1☐ 2☐ 3☐ 4☐ 5☐

10. In your opinion, how easy was it to use the instrument swap pedal?

    1☐ 2☐ 3☐ 4☐ 5☐

11. In your opinion, how easy was it to use the diathermy pedal?

    1☐ 2☐ 3☐ 4☐ 5☐

Thank you for your kind participation!
